# Medial Temporal Lobe Contributions to Intra-Item Associative Recognition Memory in the Aging Brain

**DOI:** 10.3389/fnbeh.2013.00222

**Published:** 2014-01-02

**Authors:** Marshall Axel Dalton, Sicong Tu, Michael Hornberger, John Russel Hodges, Olivier Piguet

**Affiliations:** ^1^Neuroscience Research Australia, Sydney, NSW, Australia; ^2^School of Medical Sciences, University of New South Wales, Sydney, NSW, Australia; ^3^ARC Centre of Excellence in Cognition and its Disorders, Sydney, NSW, Australia

**Keywords:** associative memory, medial temporal lobe, hippocampus, perirhinal cortex, aging

## Abstract

Aging is associated with a decline in episodic memory function. This is accompanied by degradation of and functional changes in the medial temporal lobe (MTL) which subserves mnemonic processing. To date no study has investigated age-related functional change in MTL substructures during specific episodic memory processes such as intra-item associative memory. The aim of this study was to characterize age-related change in the neural correlates of intra-item associative memory processing. Sixteen young and 10 older subjects participated in a compound word intra-item associative memory task comprising a measure of associative recognition memory and a measure of recognition memory. There was no difference in performance between groups on the associative memory measure but each group recruited different MTL regions while performing the task. The young group recruited the left anterior hippocampus and posterior parahippocampal gyrus whereas the older participants recruited the hippocampus bilaterally. In contrast, recognition memory was significantly worse in the older subjects. The left anterior hippocampus was recruited in the young group during successful recognition memory whereas the older group recruited a more posterior region of the left hippocampus and showed a more bilateral activation of frontal brain regions than was observed in the young group. Our results suggest a reorganization of the neural correlates of intra-item associative memory in the aging brain.

## Introduction

The medial temporal lobes (MTL) contain structures that are crucial for memory processing, such as the hippocampus, perirhinal cortex, and the parahippocampal gyrus. Damage to these structures invariably results in episodic memory impairment (Scoville and Milner, 1957). A growing body of evidence indicates that substructures within the MTL support different elements of episodic memory processing. The hippocampus is implicated in between-domain associative memory (Mayes et al., 2004; Konkel et al., 2008), that is the high level integration and “binding” of perceptual and conceptual information which are processed and stored in distal brain regions with weak or no direct connectivity with each other. In contrast, extra hippocampal cortical structures such as the perirhinal cortex are implicated in intra-item associative memory (Bussey et al., 2005), that is the unitization of perceptual or conceptual domains represented in closely interacting cortical regions (Davachi, 2006; Mayes et al., 2007). Additional memory functions have also been attributed to these structures. As such, it is argued that the hippocampus is crucial for recollection based recognition memory whereas the perirhinal cortex underlies familiarity based recognition memory (Mayes et al., 2007). In addition, existing evidence shows that memory processing of verbal information or information with a semantic content is lateralized to the anterior regions of the left MTL (Parsons et al., 2006; Ford et al., 2010).

Episodic memory functions decline with age (Christensen, 2001; Nyberg et al., 2003; Ronnlund et al., 2005; Schaie, 2005; Troyer et al., 2011) and young adults consistently outperform older adults on memory tasks which are hypothesized to be hippocampal dependent (Shaw et al., 2006; Head and Isom, 2010; Harris and Wolbers, 2012). MRI and *postmortem* pathological investigations have shown that among MTL substructures, the hippocampus is particularly sensitive to age-related change (Jack et al., 1998; Raz et al., 2004; Raz and Rodrigue, 2006) with the subiculum and dentate gyrus particularly affected in non-demented older adults (West, 1993; Small et al., 2002). In contrast, the perirhinal cortex appears to undergo little age-related atrophy (Insausti et al., 1998). Functional imaging studies consistently show age-related reductions in BOLD signal in the MTL during memory task performance (Daselaar et al., 2003; St Jacques et al., 2012).

Investigations of age-related change in the neural correlates of episodic memory retrieval suggest that in parallel with reductions in MTL activation, performance in older adults is associated with the recruitment of additional brain regions, often resulting in a bilateral pattern of activation in the MTL and in frontal cortical regions (Cabeza, 2001; Maguire and Frith, 2003; Giovanello and Schacter, 2012). Whether changes in brain activation as people get older are observed regardless of the type of episodic memory processes involved remains incompletely understood. It is unknown whether age-related changes in activation within and beyond the MTL are also found for specific types of associative memory processing such as intra-item associative memory. To address this issue, the present study aimed to: (1) compare intra-item associative recognition memory performance in young and older healthy adults, and (2) establish age-related changes in the neural correlates of intra-item associative recognition memory within the MTL. We used an associative memory task for compound words which included previously studied (targets) and novel items (foils). Some of the novel items were recombined elements of studied components.

We hypothesized that: (1) young adults would outperform older adults on a compound word intra-item associative recognition memory task, (2) successful intra-item associative recognition memory of compound words would recruit greater left > right MTL structures, and (3) the older group would show decreased hippocampal activity compared to the young group.

## Materials and Methods

### Participants

Sixteen, young (12 females) (mean age = 26 years, range = 21–37 years) and 10 older (5 females) (mean age = 70 years, range = 62–77 years) right-handed healthy volunteers were recruited for this study. All were native English speakers with normal or corrected to normal vision and reported no significant neurological or psychiatric disorders on a medical questionnaire. All participants underwent a cognitive screening assessment using the Addenbrooke’s Cognitive Examination-Revised (ACE-R). This study was approved by the University of New South Wales Human Ethics Research Committee and all participants provided written informed consent.

### Stimulus materials

This study comprised a verbal intra-item associative memory task, adapted from a previous publication (Mayes et al., 2007). Stimuli comprised 100 two- or three-syllable compound words (e.g., gateway, highchair). All stimuli were printed in black on a white background. Three different versions of the task were created and lists were randomly allocated across participants.

### Procedures

**Figure 1:**
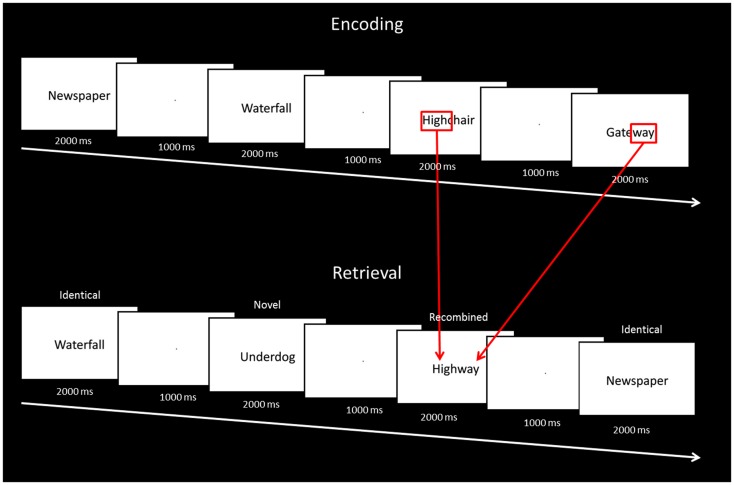
**Description of the study and test phases of the intra-item associative memory task**.

The entire experiment was conducted in the scanner and functional magnetic resonance imaging data were acquired during both encoding and test phases. All responses were recorded using an MR compatible button box. At encoding, 60 stimuli were presented on a Phillips LCD monitor one at a time in the center of the screen for 2000 ms, followed by a fixation point for 1000 ms. To ensure optimal attention to the stimuli at encoding, participants were instructed to indicate for each stimulus whether the word was pleasant, unpleasant, or neutral. Before the study phase participants were informed: “You will be shown a number of words. Please tell me if each word evokes a pleasant, unpleasant, or neutral feeling.” Encoding was immediately followed by a test phase. At test, 60 stimuli were presented one at time using the same timing procedure. Twenty stimuli were identical to the ones seen at study (“identical”), 20 were novel stimuli not seen at study (“novel”), and 20 stimuli were the combination of two stimuli seen at study (“recombined”) (e.g., *highchair* and *gateway* at encoding were recombined to become *highway* at test; Figure 1). Memory for the stimuli seen at study was tested using a yes/no recognition procedure. For each item, participants were instructed to indicate “yes” if they thought the stimulus had been presented at encoding, or “no” if they thought the stimulus had not been seen at encoding. Before the test phase participants were informed: “You will now be shown some more words. For each word please do the following. If you saw the word earlier, press the left button. If you did not see the word earlier, press the right button.” Participants were also instructed to respond within the 2000-ms stimulus presentation window. At test, the order of presentation of identical, recombined, and novel stimuli was pseudo-randomized, in that no items from the same category were seen in succession. In order to become familiarized with the general procedure, participants took part in a practice trial of the encoding and test phases outside the scanner. Participants were not informed about the recombined items at any stage of the experiment.

### MR imaging protocol

MR imaging was acquired on a 3-T Philips Achieva MRI scanner with standard quadrature head coil (16 channels). Functional MR images consisted of the following scanning parameters: 33 slices were collected per image volume covering the whole brain. Scanning parameters for the echo planar imaging (EPI) sequence were as follows: repetition time/echo time (TR/TE) 2000/30 ms; flip angle (FA) 80°; slice thickness 3.5 mm with a 0-mm interslice gap. For the current task (see below), two encoding runs were collected (75 acquisitions per run). Each encoding run was immediately followed by a test run (75 acquisitions per run). In addition, all participants underwent a whole brain T1 coronal orientation, matrix 256 × 256, 180 slices, 1 mm isotropic, TE/TR = 2.5/5.4 ms, FA α = 8°.

### fMRI data analysis

Images were analyzed using fMRI Expert Analysis Tool (FEAT) version 5.98, a part of FSL (FMRIB’s Software Library, www.fmrib.ox.ac.uk/fsl). Pre-processing of each individual’s fMRI dataset included: removal of non-brain structures from the T1 structural scans using Brain Extraction Tool (BET), motion correction using MCFLIRT, non-brain structures were removed from the echoplanar imaging volumes using BET, spatial smoothing using a Gaussian Kernel of FWHM 5 mm; mean based intensity normalization of the entire 4D dataset by the same multiplicative factor; high pass temporal filtering (Gaussian weighted least-squares straight line fitting, with σ = 100 s). Time series statistical analysis was performed using FILM with local autocorrelation correction. Functional scans were registered to the high resolution T1 structural scan per participant and to the standard Montreal Neurological Institute (MNI 152) standard space template image using affine registration with FLIRT. Coordinates (*x*, *y*, *z*) of activation are reported in MNI space.

For each subject a fixed effects model was used to estimate effects for each stimulus type. The following contrasts were modeled: identical item hits vs. correct novel item rejections and identical item hits vs. correct recombined item rejections. The resulting data were then entered into a mixed effects higher level analysis to investigate activity across participants for each comparison. *Z* statistic images were thresholded using clusters determined by *Z* > 2.3 and an uncorrected cluster significance threshold of *p* < 0.01. In addition, the % signal change within each cluster was extracted for each group.

## Results

### Behavioral

**Figure 2:**
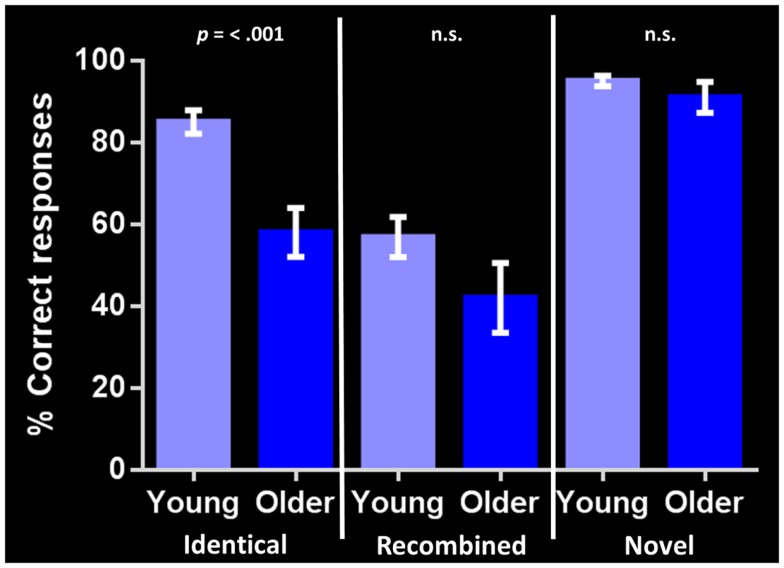
**Corrected recognition memory performance for each stimulus condition in young and older adults (i.e., hits-false alarms)**. The bar chart shows the percent correct response for each stimulus type (identical, recombined, novel). Error bars represent the standard error of the mean. *p* = < 0.001 indicates significant difference between young and older adult groups. n.s., indicates non-significant difference.

Performance on the general cognitive measure ACE-R was not significantly different between the young and older groups. Corrected recognition memory for identical compound words (i.e., hits-false alarms) differed significantly between groups, with the young participants outperforming their older counterparts [85 and 58% respectively; *t*(24) = 4.568, *p* = < 0.001]. In contrast, no significant group differences were found in identifying either *recombined* [57 and 42% respectively; *t*(24) = 1.630, *p* = 0.116] or *novel* compound words [95 and 91% respectively; *t*(24) = 1.182, *p* = 0.249] (Figure 2). Investigating within group performance, we found significant differences in accuracy between identical and recombined items [85 vs. 57%, *t*(30) = 4.951, *p* = < 0.001], between recombined and novel items [57 vs. 95%, *t*(30) = −7.535, *p* = < 0.001], and between identical and novel items [85 and 95%, *t*(30) = −3.162, *p* = 0.004] in the young group. In contrast, in the older group, there was a significant difference in accuracy between recombined and novel items [42 and 91%, *t*(18) = −5.247, *p* = < 0.001] and identical and novel items [58 and 91%, *t*(18) = −4.693, *p* = < 0.001] but no significant difference between identical and recombined items [58 and 48%, *t*(18) = 1.540, *p* = 0.141].

Analyses on response latency revealed that in the young group, correct responses to recombined items (1160 ± 271 ms) were significantly slower than responses to identical [1003 ± 243 ms, *t*(681) = −7.96, *p* = < 0.001] and novel [1008 ± 262 ms, *t*(683) = 7.43, *p* < 0.001] items. No significant difference was found in response latency between identical and novel items. In the older group, correct responses to recombined items (1246 ± 285 ms) were significantly slower than responses to identical [1112 ± 280 ms, *t*(332) = −4.327, *p* = < 0.001] and novel [1084 ± 280 ms, *t*(334) = 5.233, *p* = < 0.001] items. No significant difference was found in response latency between identical and novel items.

The young group responded significantly faster than the older group for all item types: identical [young: 1003 ± 243 ms; older: 1112 ± 278 ms; *t*(521) = −4.6, *p* = < 0.001], recombined [young: 1159 ± 271 ms; older: 1246 ± 285 ms; *t*(494) = −3.3, *p* = < 0.001], and novel [young: 1008 ± 262 ms; older: 1084 ± 280 ms; *t*(523) = −3.08, *p* = 0.002]. The number of late responses (i.e., >2000 ms) did not differ between the young and older groups.

### Imaging

#### Associative recognition memory (identical vs. recombined contrast)

**Figure 3:**
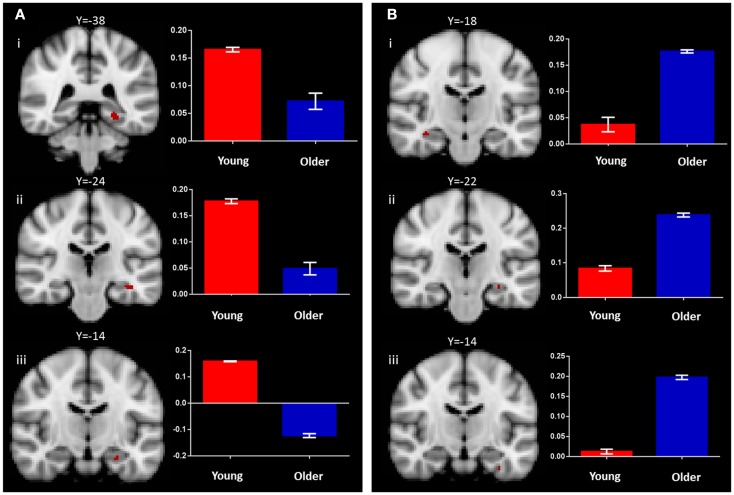
**Regions of increased BOLD signal associated with associative recognition memory (identical > recombined rejection) for: (A) young: (i) left posterior parahippocampal gyrus (*x* = −26, *y* = −38, *z* = −10); (ii,iii) left anterior hippocampus (*x* = −36, *y* = −24, *z* = −14) (*x* = −26, *y* = −14, *z* = −22) and (B) old: (i) right anterior hippocampus (*x* = 36, *y* = −18, *z* = −16); (ii) left anterior hippocampus (*x* = −30, *y* = −22, *z* = −14); (iii) left anterior parahippocampal gyrus (*x* = −32, *y* = −14, *z* = −32) subjects**. Graph depicts mean percent signal change associated with each group within the region of interest.

In the young group, the identical vs. recombined rejection contrast revealed significant activation within the MTL in two regions of the left hippocampus (*x* = −36, *y* = −24, *z* = −14 and *x* = −26, *y* = −14, *z* = −22) and in the left posterior parahippocampal gyrus (*x* = −26, *y* = −38, *z* = −10) (Figure 3A; Table 1). In addition, significant clusters of activation were also observed in the middle frontal gyrus, putamen, lateral occipital cortex, precentral gyrus, posterior cingulate gyrus, supramarginal gyrus, and frontal pole bilaterally (Table 1).

**Table 1:** **BOLD signal increase for the contrast of identical hits > correct recombined rejection in young and older participants**.

	Regions	Hemisphere (L/R/B)	MNI coordinates			Number of voxels	*t*-Score
			*X*	*Y*	*Z*		
Young	Middle frontal gyrus	L	−40	20	44	927	3.7
	Putamen	R	28	−6	−2	884	3.51
	Juxtapositional lobule cortex	R	6	−10	52	841	3.67
	Lateral occipital cortex	L	−50	−64	38	725	3.53
	Frontal pole	R	40	52	−8	330	3.29
	Frontal pole	L	−20	50	28	253	3.14
	Frontal pole	L	−42	46	6	219	3.11
	Cerebellum	L	−16	−58	−22	208	3.08
	Precentral gyrus	R	60	6	4	101	2.88
	Posterior cingulate gyrus	R	2	−38	2	82	2.93
	Supramarginal gyrus	L	−56	−42	30	71	2.62
	Frontal pole	L	−22	62	4	67	2.82
	Cerebellum	L	−2	−66	−20	56	2.93
	Putamen	L	57	69	34	50	2.72
Young MTL	Posterior parahippocampal gyrus	L	−26	−38	−10	33	2.82
	Hippocampus	L	−26	−14	−22	17	2.65
	Hippocampus	L	−36	−24	−14	13	2.74
Older	Insular cortex	L	−32	−12	14	132	2.91
	Posterior cingulate gyrus	R	8	−24	46	71	2.74
	Occipital fusiform gyrus	L	−20	−68	−16	64	2.76
	Middle frontal gyrus	R	28	28	30	59	2.7
Older MTL	Hippocampus	R	36	−18	−16	10	2.96
	Hippocampus	L	−30	−22	−14	3	2.32
	Anterior parahippocampal gyrus (perirhinal)	L	−32	−14	−32	2	2.24

*Results are reported at *p* < 0.001 uncorrected with at least 50 contiguous voxels*.

The same contrast in the older group revealed significant activation within the MTL in the hippocampus bilaterally (right: *x* = 36, *y* = −18, *z* = −16 and left: *x* = −30, *y* = −20, *z* = −14) and the left perirhinal cortex (*x* = −32, *y* = −14, *z* = −32) (Figure 3B; Table 1). Additional significant activation was observed in the insular cortex, posterior cingulate cortex, occipital fusiform gyrus, and the middle frontal gyrus (Table 1).

#### Recognition memory (identical vs. novel contrast)

**Figure 4:**
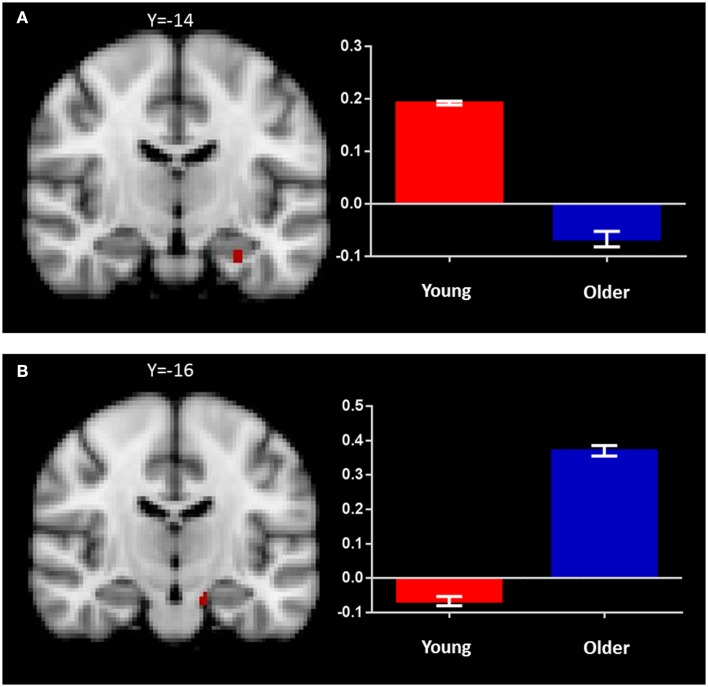
**Regions of increased BOLD signal associated with recognition memory (identical > novel) for: (A) young: left anterior hippocampus (*x* = −28, *y* = −14, *z* = −24) and (B) old (*x* = −14, *y* = −16, *z* = −22) subjects**. Graph depicts mean percent signal change associated with each group within the region of interest.

In the young adult group, the identical vs. novel item contrast revealed a significant cluster of activation in the left anterior hippocampus (*x* = −28, *y* = −14, *z* = −24; Figure 4A; Table 2). In addition, significant activation in were found in the lateral occipital cortex, middle frontal gyrus, frontal pole, thalamus, superior frontal gyrus, and precuneus (Table 2).

**Table 2:** **BOLD signal increase for the contrast of identical hits > novel hits in young and older participants**.

	Regions	Hemisphere (L/R/B)	MNI coordinates			Number of voxels	*t*-Score
			*X*	*Y*	*Z*
Young	Lateral occipital cortex	L	−46	−64	42	667	3.39
	Middle frontal gyrus	L	−46	18	34	274	3.16
	Frontal pole	R	44	36	20	138	3.03
	Lateral occipital cortex	R	36	−72	46	126	2.74
	Thalamus	R	12	−10	4	121	2.82
	Superior frontal gyrus	R	26	30	54	71	2.95
	Juxtapositional lobule cortex	R	6	−8	48	70	3.19
	Frontal pole	R	10	56	18	68	2.72
	Precuneus cortex	R	4	−56	34	61	2.64
Young MTL	Hippocampus	L	−28	−14	−24	14	2.67
Older	Angular gyrus	L	−50	−56	48	846	3.05
	Superior frontal gyrus	L	−20	20	56	622	3.04
	Lateral occipital cortex	R	40	−64	38	447	3.0
	Middle frontal gyrus	R	36	10	48	245	2.9
	Frontal pole	L	−38	40	6	207	2.89
	Middle frontal gyrus	L	−52	30	24	201	2.86
	Frontal pole	L	−10	64	20	148	2.93
	Middle frontal gyrus	R	44	26	24	83	2.81
	Frontal pole	R	20	52	12	82	2.72
	Precentral gyrus	R	46	4	26	79	2.82
	Frontal pole	L	−18	66	0	76	2.89
	Paracingulate gyrus	L	−12	44	14	64	2.63
	Supramarginal gyrus	L	−60	−46	40	50	2.7
Older MTL	Hippocampus	L	−14	−16	−22	7	2.44

*Results are reported at *p* < 0.001 uncorrected with at least 50 contiguous voxels*.

In the older group, the same contrast revealed a small cluster of activation within the left hippocampus (*x* = −14, *y* = −16, *z* = −22; Figure 4B; Table 2). Broad bilateral activation was also present in the middle frontal gyrus and the frontal pole, as well as in the angular gyrus, superior frontal gyrus, lateral occipital cortex, precentral gyrus, paracingulate gyrus, and the supramarginal gyrus (Table 2).

## Discussion

This study identified the neural correlates of associative recognition memory for compound words in young and older healthy adults. Although performance in associative recognition memory was matched between groups, young and older adults differed in the location and extent of MTL involvement supporting task performance. Young adults showed a left lateralized activation involving the anterior hippocampus and posterior parahippocampal gyrus. In contrast, older participants revealed hippocampal involvement bilaterally. In addition to these MTL regions, associative memory performance was also associated with increased activity in a number of cortical and subcortical regions including the middle frontal gyrus, putamen, lateral occipital cortex, precentral gyrus, posterior cingulate gyrus, supramarginal gyrus, and frontal pole in the young adults, as well as the insular cortex, posterior cingulate, occipital fusiform, and middle frontal gyrus in the older adults.

These results suggest a reorganization in the neural correlates of associative recognition memory for compound words with age. Imaging findings from the young group align well with the view that verbal memory processing is supported by a left lateralized distributed network involving anterior regions of the MTL (Binder et al., 2003; Daselaar et al., 2003; Parsons et al., 2006; Ford et al., 2010). More specifically, we found left anterior hippocampal and posterior parahippocampal gyrus activation in this group. A previous study utilized a similar task to the one used in the present study to investigate associative memory processing in young adults and reported recruitment of the left perirhinal cortex (Ford et al., 2010). We found no evidence of left perirhinal cortex recruitment in the young group but did observe a small left perirhinal cortex cluster in the older group. Importantly, bilateral hippocampus recruitment was also present in the older group. To our knowledge, this is the first observation of age-related functional change in the neural correlates of associative recognition memory for compound words. Age-related changes affecting the laterality of MTL activation have previously been observed in functional imaging studies of autobiographical memory retrieval, with predominant left hippocampus recruitment found in young adults compared to bilateral activation in older adults (Maguire and Frith, 2003). We observed a similar age-related left–right shift in MTL activation during successful associative recognition memory for compound words. Involvement of the left anterior hippocampus was observed only in the young group. In the older group, activation of more posterior regions of the hippocampus bilaterally supporting memory performance was found instead.

In addition to changes in MTL activity, we also observed changes in a number of cortical and subcortical brain regions, which have been previously implicated in verbal memory processing, verbal fluency, and naming of objects (Valenstein et al., 1987; Petrides et al., 1993; Salmon et al., 1996; Rosen et al., 2000; Chouinard et al., 2009; Bokde et al., 2010; Lim et al., 2012; Thames et al., 2012; Costa et al., 2013). Recruitment of some of these regions was age specific. Activation in the putamen, frontal pole, supramarginal gyrus, and lateral occipital cortex was found in the young group only. Regions of activation observed only in the older group included the insular cortex and fusiform gyrus, again regions that have been previously implicated in verbal memory (Paulesu et al., 1993; Grasby et al., 1994; Manes et al., 1999) although the fusiform cortex is generally considered to be involved in memory processing of non-verbal pictorial rather than verbal stimuli (Kim, 2011).

In contrast to associative recognition memory, recognition memory (i.e., correct identification of identical stimuli) differed between groups, with older adults experiencing greater difficulty than young participants on this component of the task. As anticipated, the neural correlates of recognition memory also differed between groups. During this task, young adults recruited the left anterior hippocampus whereas older adults showed involvement of the left hippocampus more posteriorly with additional recruitment of frontal cortical regions bilaterally. In other words, older adults were unable to maintain a level of performance similar to that of young adults despite the recruitment of additional brain regions. The increase in bilateral brain activation with age during memory retrieval has been reported previously and appears to reflect a compensatory process (Reuter-Lorenz and Cappell, 2008; Cappell et al., 2010). Although recognition of recombined stimuli is inherently more difficult than that of identical stimuli, we found an age difference on the recognition performance for the identical but not the recombined component of the task. Whilst we observed a drop in response accuracy between identical and recombined stimuli for the young group, performance in the older group between conditions remained relatively unchanged. Two explanations may underlie this unanticipated result. First, evidence indicates that older adults tend to show a more liberal response pattern in recognition memory tasks compared to young adults (Huh et al., 2006). Frequency of false alarms during recognition memory tasks also tends to rise with task difficulty, regardless of age. Indeed, in this study, the young group showed higher false alarms in the recombined than the identical conditions. The frequency of false alarms, however, remained stable in the older group. This may have been due to the task instructions. Here, correct responses to the instructions (“Have you seen this word before?”) necessitated opposing behaviors depending on the stimulus types: “yes” responses to identical stimuli, but “no” responses to recombined stimuli. It is therefore plausible that the response type required in this condition (i.e., having to reject correctly identified recombined stimuli) may have counterbalanced the liberal bias generally observed in the older group and reduced the false alarm responses, thus reducing the between-group difference. Second, close inspection of individual response profiles revealed that three young participants scored at least 2 SDs below the group mean for the recombined condition, contributing to the lack of group difference^1^.

The group difference in the patterns of brain activation may represent a functional reorganization and compensation for the decreased efficiency in hippocampal recruitment found as individuals get older. Hippocampal volumes decrease with healthy aging (Raz and Rodrigue, 2006) and are predictive of explicit memory performance in subjects over the age of 60 (Raz et al., 1998; Lye et al., 2004). Loss of synaptic density, rather than neuronal loss, is the main contributor to the volume reduction (Rosenzweig and Barnes, 2003; Burke and Barnes, 2006). Not all hippocampal regions undergo the same changes, however, with anterior regions appearing to be more resilient to age-related degradation than posterior regions (Driscoll et al., 2003). Within this framework, the recruitment of posterior regions of the hippocampus in the older group remains to be investigated further. Age-related reductions in hippocampal activation have been reported during a number of memory tasks using differing methodologies such as encoding of nouns (Daselaar et al., 2003), autobiographical memory retrieval (St Jacques et al., 2012), working memory for complex scenes (Park et al., 2003) and relational encoding in working memory (Mitchell et al., 2000). In addition, memory performance is also affected by the integrity of the connections between MTL and surrounding structures (Hornberger et al., 2012). The reduced activation in the left anterior hippocampus found in the older group accords well with these findings.

The perirhinal cortex and parahippocampal gyrus are components of two dissociable cortical networks with the perirhinal cortex contributing to memory for item information and the parahippocampal gyrus contributing to memory for context (Ranganath and Ritchey, 2012). The parahippocampal gyrus has also been implicated in episodic simulation (Addis et al., 2009). As such, the difference in brain activation may also indicate the use of different mnemonic strategies in the young and older groups. The increased posterior parahippocampal gyrus activation and lack of perirhinal cortex activation in the young group possibly reflects the reliance on mnemonic techniques such as visualization or mental elaboration in this group (e.g., Kondo et al., 2005). In post task debriefings, participants commonly mentioned the use of visualization and association as mnemonic tools to help remember each item. Whether the use of such strategies differed between the two groups was not formally investigated.

Arguably, our findings need to be taken with caution in the view of the uncorrected results reported here. Nevertheless, while the activation clusters in the MTL may appear small, they are in line with those found previously (Staresina and Davachi, 2006). These potential limitations notwithstanding, we have shown that associative recognition memory for compound words is associated with left lateralized MTL structures in young individuals and bilateral MTL structures in their older counterparts. We provide evidence for a functional reorganization of the neural correlates of associative memory processing in the aging brain. These findings have important implications for theoretical models of associative memory processing, in that they support the view that different regions of the MTL are capable of supporting associative recognition memory for verbal stimuli in different stages of life.

## Conflict of Interest Statement

The authors declare that the research was conducted in the absence of any commercial or financial relationships that could be construed as a potential conflict of interest.

## Footnotes

^1^Indeed, re-analysis of the behavioral data when these three outliers from the young group are excluded result in a significant between-group difference for the recombined condition.

## Appendix

**Table A1:** **Example list of words used in the intra-item associative memory task**.

Study		Test		
		Identical	Recombined	Novel
**WORDS USED AS RECOMBINED ITEMS AT TEST**				
Bluebell	Hummingbird	Sandstorm	Bluebird	Underground
Postcard	Trademark	Snowman	Postmark	Sunlight
Playroom	Checkmate	Needlepoint	Playmate	Lapdog
Fireboat	Marketplace	Artwork	Fireplace	Spacecraft
Fatherland	Neighborhood	Meatball	Fatherhood	Jumpsuit
Downstairs	Waterfall	Brainpower	Downfall	Headdress
Earthmover	Silkworm	Cupcake	Earthworm	Eyewitness
Mainframe	Slipstream	Shuffleboard	Mainstream	Foresight
Newspaper	Bandstand	Handbag	Newsstand	Keystone
Outlook	Trapdoors	Horserace	Outdoors	Crossword
Stardust	Bracelet	Blowtorch	Starlet	Turntable
Nightshade	Hubcap	Sidewalk	Nightcap	Breakfast
Facemask	Forklift	Smokestack	Facelift	Getaway
Beehive	Housekeeper	Flyscreen	Beekeeper	Armrest
Hedgerow	Warthog	Boxcar	Hedgehog	Coalpit
Backbone	Raindrop	Footstep	Backdrop	Hourglass
Bottleneck	Paintbrush	Buttonhole	Bottlebrush	Spymaster
Steamroller	Longboat	Motorcycle	Steamboat	Drumbeat
Shortcut	Gingerbread	Courtship	Shortbread	Waistcoat
Skydive	Mudlark	Vineyard	Skylark	Joystick
**WORDS USED AS IDENTICAL ITEMS AT TEST**				
Sandstorm	Buttonhole			
Snowman	Boxcar			
Needlepoint	Courtship			
Artwork	Blowtorch			
Meatball	Footstep			
Brainpower	Vineyard			
Cupcake	Sidewalk			
Shuffleboard	Smokestack			
Handbag	Motorcycle			
Horserace	Flyscreen			
